# The Performance Impact of New Ventures in Working Environment and Innovation Behavior From the Perspective of Personality Psychology

**DOI:** 10.3389/fpsyg.2021.734014

**Published:** 2021-11-03

**Authors:** Shufang Yang, Hainan Wu

**Affiliations:** ^1^School of Business, Changshu Institute of Technology, Suzhou, China; ^2^School of Finance and Public Administration, Anhui University of Finance and Economics, Bengbu, China

**Keywords:** personality psychology, new venture, work environment, innovation behavior, corporate performance, teamwork, organizational encouragement, work pressure

## Abstract

A new venture barely makes a profit in its initial stage, and its success depends on innovation. Innovation is related to the work environment, and the innovation behavior of employees is of great significance to the performance improvement of new venture. Based on the previous research, in this study, hypotheses on the correlation between work environment, employee innovation behavior, and corporate performance are put forward first. Then, with team cooperation, organizational incentive, leadership support, sufficient resources, and work pressure as the factors of the work environment, the bosses, middle and senior managers involved in entrepreneurship, and the main members of the entrepreneurial team of 202 newly established enterprises in developed regions are surveyed online or in scene. Multivariate hierarchical regression analysis is performed to analyze the data collected from the questionnaire. The results show that the effective recovery rate of the questionnaire is 86.4%; the number of traditional enterprises is 108 (53.47%), and that of R&D enterprises is 68 (33.66%); teamwork, leadership support, and work pressure are all correlated with employees’ innovative behavior (*P* < 0.05), while organizational motivation and sufficient resources are not correlated with employees’ innovative behavior (*P* > 0.05); employee innovation behavior is positively correlated with enterprise performance (β = 0.375, *P* ≤ 0.01); the working environment and employee innovation behavior promote enterprise performance (β = 0.433, *P* ≤ 0.01); and the working environment affects the relationship between employee innovation behavior and enterprise performance (β = 0.399, *P* ≤ 0.05). The study theoretically enriches the research on the relationship between innovation behavior, work environment, and enterprise performance of new ventures. In practice, it is suggested that start-up enterprises provide good working environment for employees and attach importance to innovation activities at the individual level of employees, which provides useful guidance and reference for the development of Chinese start-up enterprises.

## Introduction

Usually, personality psychology is used to study individual behavior patterns. It can predict the impact of the composition characteristics, formation methods, and influencing factors of individuals or groups on human behavior (Southward and Cheavens, 2020). Innovation and entrepreneurship are the main forces driving social and economic development, and the success of an enterprise depends on innovation. Innovation behavior is a synthesis of all behaviors taken by individuals in the generation, practice, promotion, and application of new ideas. As the source of innovation ideas, employees are also the implementers of innovation activities (Zhang and Su, 2020). Innovation behavior of employees can not only improve their own work efficiency, but also promote the company’s development (Li C. et al., 2020). Studies have pointed out that employee innovation has a significant impact on corporate innovation (Hameed et al., 2021). It is believed that the individual’s sense of innovation determines the creativity of the entire organization (Bishwas and Kumar, 2015). Therefore, the innovation behavior of employees will determine the reform of the enterprise’s products and services. However, most of the research on creativity is focused on college students, and there are few studies on enterprise innovation.

Employee innovation behavior is related to the employee creativity. Creativity in organizations is different from other ideas and things, and it will affect the long-term or short-term behavior (Wallace et al., 2016). Employee creativity refers to the creation of novel and potentially valuable things or ideas, including new products, services, manufacturing methods, and management processes, which can promote the survival, innovation, and growth of enterprises in the fierce competition (Odoardi et al., 2019). Innovation in the traditional sense mainly focuses on human factors, and it is believed that innovation is determined by people’s innovation quality, although later studies have found that factors such as creative background, personality traits, and working mode also affect innovation behavior (Hu and Zhao, 2016). At the same time, some studies have pointed out that the factors promoting innovation mainly include: freedom, encouragement, challenge, identity and feedback, sufficient time, sufficient resources, appropriate pressure, good project, and positive organizational characteristics; and that factors that hinder creativity mainly include: constraints, insufficient resources, lack of time pressure, inappropriate evaluation, lack of organization and enthusiasm, bad projects, negative organizational characteristics, and over-emphasis on competitive status (Khan and Khan, 2019). Subsequent studies have found that factors such as creative background, personality and working style have a certain impact on individual’s innovativeness (Li et al., 2017), but there are still some limitations.

Working environment refers to the surrounding conditions that have an impact on manufacturing process and product quality. Agricultural enterprises refer to the integrated services of production, processing, storage, sales, production and sales. They are engaged in the design of agricultural commodities, with high commodity rate and independent operation (Shevchenko et al., 2019). They are economic entities concerning agricultural production, agricultural products processing, and agricultural production services. They mainly provide pre-production, in-production and post-production services for agricultural production, including processing enterprises, enterprises directly engaged in animal husbandry and fishery production with agricultural products as raw materials, indirect and agriculture-related enterprises and agricultural intermediary, agricultural information and agricultural science and technology enterprises. The special functions of agriculture-related enterprises are mainly reflected in: (1) providing the most basic material materials for the society; (2) producing a variety of agricultural and sideline products and processed products for production and processing; (3) protecting the natural ecological environment system; and (4) improving the organizational degree of farmers. With the globalization of economy and information, the competition among enterprises is becoming more and more fierce, and the competitiveness of enterprises increasingly depends on their innovation ability (Kitouni et al., 2018). In a changing environment, enterprise innovation is not only to adapt to the environment, but also inevitably affected by the working environment (Yang et al., 2016). Researchers have pointed out that the work environment is a dynamic process, mainly involving team cooperation, leadership support, working atmosphere, and resource allocation, which are all related to the creativity of enterprises (Chen, 2019). Some researchers conducted research on the relationship between superiors and subordinates, organizational goals, work structure, organizational structure, colleague relationship, autonomy, management support, internal conflict, performance standards, and communication frequency, among which the organizational atmosphere scale is the most influential (Riyanto et al., 2017). Morrell et al. (2019) proposed to measure innovation, team building, planning responsibility, and inertia tendency. Saidi et al. (2019) put forward a table of work environment, which mainly includes encouragement, independence, resources, pressure, and organizational obstacles.

In recent years, with the continuous development of e-commerce, new ventures spring up like mushrooms. However, the average life span of domestic small and medium enterprises (SMEs) is only 3–5 years, which is not optimistic (Jantz, 2015). New venture refers to the enterprise that is still in the development stage and has not been mature. Studies have found that an enterprise going through the first 6 years can basically survive, and thus the first 6 years are used as the boundary line (Deligianni et al., 2017). Some scholars hold that 7 years are used as the boundary line because most new ventures can achieve stable profitability after 7 years (Gegenhuber and Dobusch, 2016). There is also an idea that all companies established for less than 8 years should be divided into the category of new venture (Ter Wal et al., 2016).

The enterprise performance is a quantitative indicator to evaluate the operation of an enterprise. Generally, performance evaluation involves financial performance and growth performance (Pouvaret et al., 2020). Financial indicator alone can’t comprehensively measure the performance of new ventures, because new venture rarely makes a profit in the initial stage thanks to fierce market competition (Qian et al., 2018). Therefore, some scholars suggest that growth indicators be incorporated to evaluate the performance of new ventures. Financial indicators are objective but confidential (Haghshenas et al., 2021), and thus subjective evaluation methods are currently used (Khoshnevis and Teirlinck, 2017). Nevertheless, the current research still has the following shortcomings. It still remains unclear whether organizational encouragement, superior support, teamwork, adequate resources, and work stress will affect the innovation behavior of employees, especially under the increasingly fierce competitive environment. Additionally, the correlation between work environment and innovation behavior with corporate performance remains to be explored (Feng and Chen, 2020).

### Research Model and Research Hypothesis

The individual’s innovation behavior is affected personal knowledge, intentions, ability, and the environment affects, alone or in combination. Lee et al. (2020) studied the impact of work environment on the corporate rent based on Amabile’s creativity theory, and found that work environment is associated with corporate rents. Taohid et al. (2021) proposed six mutually independent working environment dimensions: attention to detail, innovation ability, work independently, dealing with unfriendly people, social skills, and competition. Merga and Fufa (2019) believed that faced with the need for strict compliance and implementation, the employee is easy to feel greater pressure, reducing his/her intrinsic motivation of innovation. The theoretical innovation part of Nayak et al. (2011) focused on work motivation, and later included social environment, pointing out that environmental factors had an impact on work motivation. This innovation theory is at the individual level, leading to organizational creativity and innovation component theory (components of organizational creativity and innovation). The basic components of organizational creativity and innovation theory are the factors that influence individual creativity in the work environment, and the output of individuals or teams is the basis of organizational innovation (Micheli et al., 2019; Deng et al., 2021). The cultivation of creativity requires a free and open environment, and whether the organization or school environment of invention can prove this (Hu et al., 2018). Taking Amabile creativity theory as the basis, some researchers analyzed the work environment characteristics of comprehensive agricultural enterprises to charge rent. It was found that, these enterprises can establish the rent (the company’s overall performance was expected to surpass the past and its competitors, and its sales were expected to be more than the competitors). During the work, supervisors supported and encouraged creative problem-solving, and provided adequate resources to support employees’ work (Karjaluoto et al., 2019). Based on the above research, this study took organizational encouragement, leadership support, teamwork, sufficient resources, and work pressure as independent variables of the work environment.

**FIGURE 1:**
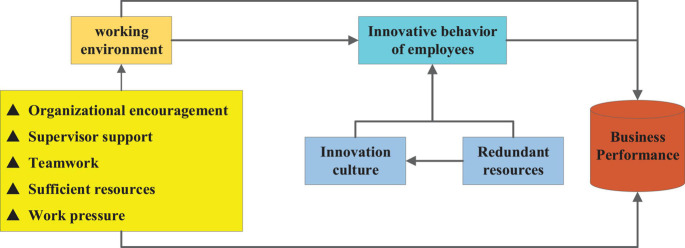
The theory model in the study.

In recent years, with the continuous development of economy and the constant change of customer demand, the competition among enterprises is becoming fiercer. The innovation output of employees directly affects the overall innovation performance of enterprises (Udriyah et al., 2019). The innovation theory believes that technological innovation has great influence on enterprise success and enterprise performance. For an enterprise, the technological innovation should make a positive contribution to its performance. Some researchers studied the correlation between innovation behavior and innovation performance of 213 industrial enterprises. The results showed that the innovation performance was closely associated with the innovation capacity; research and development ability, learning ability, and technical innovation behavior can increase the innovation performance continues, while production capacity, organization ability, resource allocation, and strategic planning ability had little effect on innovation performance (Fernando et al., 2019). Based on current research, this study takes organizational encouragement, leadership support, team cooperation, sufficient resources, and work stress as independent variables of the work environment (Merga and Fufa, 2019). The work environment factors related to innovation mainly include organizational encouragement, superior support, teamwork, adequate resources, and work stress. In theory, the innovation output of enterprise employees directly affects the overall innovation performance of the enterprise. The social cognitive theory and organizational culture theory say that the work environment will have a certain impact on individual behavior. Some researchers believe that if employees of a company can perceive that the company attaches great importance to innovation, they will make great efforts to innovate at work to promote the overall innovation of the company (Haseeb et al., 2019). Above, a theoretical model is established, as shown in Figure 1.

The factors affecting the creativity are complicated. Kim and Choi (2017) pointed out that, creativity is significantly correlated with individual independent creativity and the complexity of research tasks. At the same time, studies have pointed out that, there is a significant correlation between employee enthusiasm and team creativity (Pitafi et al., 2020). The introduction of new ideas and the inclusion of new technologies have a positive effect on the development of a new venture. Based on previous studies, the following hypotheses are put forward.

H1: Employee innovation behavior is significantly correlated with organizational encouragement, superior support, teamwork, adequate resources, and work stress in the category of work environment;

H2: Employee innovation behavior positively affects corporate performance;

H3: Work environment has a significant correlation with corporate performance;

H4: Work environment and employee innovation behavior promote corporate performance;

H5: Work environment promotes the relationship between employee innovation behavior and corporate performance.

### Research Design

In this study, new ventures are used as the research subjects, which were established in the last 8 years. The questionnaire survey method is used to collect the required data. SPSS20.0 is used to process the data, and the scales used mainly include: personal basic information, work environment scale and innovation behavior scale. At the same time, this study measures the performance of new ventures from the two aspects of profit performance and growth performance, referring to the relevant content of the scale evaluating the working environment of enterprises in the study of Gasparino and Guirardello (2017), and some modification are made by using “+” to indicate a positive factor that stimulates innovation behavior, and “−” to indicate a negative that hinders innovation behavior factor. The main content of the scale includes: (I) Encouraging innovation: The support of the organization, supervisor, and team; (II) Organizational incentives: Employees are given commendations and rewards for innovation; (III) Autonomy or freedom: A high degree of autonomy of individuals is conducive to innovation; (IV) Resources: The reasonable allocation of resources will have a certain impact on innovation behavior; and (V) Work stress: Involving challenging work and workload pressure (Wu and Song, 2019). The scale factors into internal consistency, test-retest reliability, convergence validity, and discriminative validity. It involves 22 questions, concerning “encourage” and “superior support,” “team,” “resources,” and “work stress.”

Performance is qualitatively evaluated using “good,” “fair,” and “poor,” referring to the research of Haseeb (2019), as shown in Table 1. The 5-point scoring is adopted. A score greater than 4 is considered good; a score between 3 and 4 points, is considered fair, and a score ≤3 is considered poor.

**TABLE 1:** Corporate performance scale.

**Number**	**Question**
A1	High net yield
A2	High return on investment
A3	The company’s total sales are growing fast
A4	Rapid growth in net income
A5	Many new businesses are developed
A6	Increase in the number of new businesses
A7	Increase in net income from new business
A8	The company’s market share increases

The employee innovation behavior is evaluated referring to the scale formulated by Qi (2019), and profitability performance and growth performance are mainly included. There is a total of 12 measurement items, and the Cronbach’s value is 0.812, as shown in Table 2.

**TABLE 2:** Measurement indicators of innovation behavior.

**Number**	**Question**
B1	Employees will look for opportunities to improve the efficiency and performance of the company or department
B2	Employees will pay attention to infrequent problems at work
B3	Employees will put forward ideas or solutions to problems in the company
B4	Employees propose corresponding advantages and disadvantages of the company’s new plan
B5	Employees will risk supporting new solutions
B6	Employees will find ways to correct the shortcomings of the new plan
B7	Employees will use new solutions in their work to improve work efficiency
B8	Employees persuade others to understand the importance of new solutions

### The Questionnaire Survey

Whether questionnaire survey is used correctly, reliability, and research value are very important. Therefore, questionnaire should be issued after pre-examination preparation (pretest). The first formal pre-examination of sample feature selection was carried out from January 5, 2017 to December 20, 2018, and three agriculture-related enterprises were selected as the subjects of pre-examination, to verify the reliability and validity of the questionnaire. There was a total of 50 questionnaires, of which 1 was damaged or deleted by the interviewees in the process of filling in the questionnaire. A total of 49 valid questionnaires were collected with an effective recovery rate of 98%. After factor analysis, the items whose reliability coefficient was less than 0.6 were deleted, and the Cronbach’s α values of the other factors were all above 0.8, so they were all retained. The Cronbach’s α value of the last factor also reached 0.7, so it was also retained, as shown in Table 3.

**TABLE 3:** Details of the questionnaire.

**Factor**	**Number**	**Item**
Organizational encourage	1	The company encourages creative problem solving
	2	The company is engaged in creative work
	3	The company has a sound mechanism to encourage employees to innovate
	4	The company organizes activities to stimulate employees’ creativity
Leadership support	1	The leader is a good example in work
	2	The leader formulates an appropriate goal
	3	The lead measures members’ contributions
	4	The leader supports the work of members
	5	The leader has confidence in member innovation
Team cooperation	1	The team has open communication channels
	2	Employees exchange new ideas with each other
	3	The leader gives challenging work advice through meetings
	4	Members of the group help each other and trust each other
Sufficient resources	1	Sufficient information
	2	Sufficient funds
	3	Sufficient equipment
Working pressure	1	Challenging work
	2	The employer can solve problems in the work by his own
Innovative behavior	1	The employer always finds new ways to solve the problem
	2	The employer finds ways to get the resources needed to implement new ideas
	3	A reasonable time schedule is designed
	4	Having innovative behavior

The questionnaires in this study were mainly distributed to new ventures in Zhejiang, Anhui, Shanghai, Hangzhou, Beijing, Shenzhen, and other cities. The subjects of the questionnaire survey were mainly the bosses, middle and senior managers, and the main members of the entrepreneurial team in new ventures. Both online surveys and on-site surveys were performed. On-site surveys selected places where new ventures are more concentrated, such as technology incubators, college student entrepreneurship parks, and creative design parks. The questionnaires were issued online through professional websites. All the personnel who issued the questionnaire were trained in advance. After data analysis, the regions to be investigated is determined. Then, relevant companies that meet the requirements are selected based on the local public company directory. Next, the personnel inform the companies of their investigation intentions through emails and telephones, and asks whether the companies are willing to participate in this survey. Finally, the questionnaires are distributed online and on-site.

A total of 684 questionnaires were issued this time, 619 questionnaires were recovered, and 591 questionnaires were effectively recovered, with an effective recovery rate of 86.4%. A total of 202 companies were involved.

### Statistical Analysis

In this study, statistical product and service solutions (SPSS) is used as the data analysis tool, and all data are from the feedback data of the questionnaire. Internal consistency is an indicator to measure the correlation between a variable and other variable. It is generally expressed by the internal consistency coefficient (Cronbach’s α). A larger Cronbach’s value indicates higher reliability. Cronbach’s α value greater than 0.7 is generally considered as high reliability (Wu Y. et al., 2020). Pearson correlation analysis is used to explore the relationship between working environment, employee innovation behavior, and enterprise performance. Kaiser-Meyer-Olkin (KMO) and Bartlett’s sphericity test are used for validity analysis, and the critical value of KMO is 0.5 (Rogoza et al., 2018).

### Individual Basic Characteristics

**FIGURE 2:**
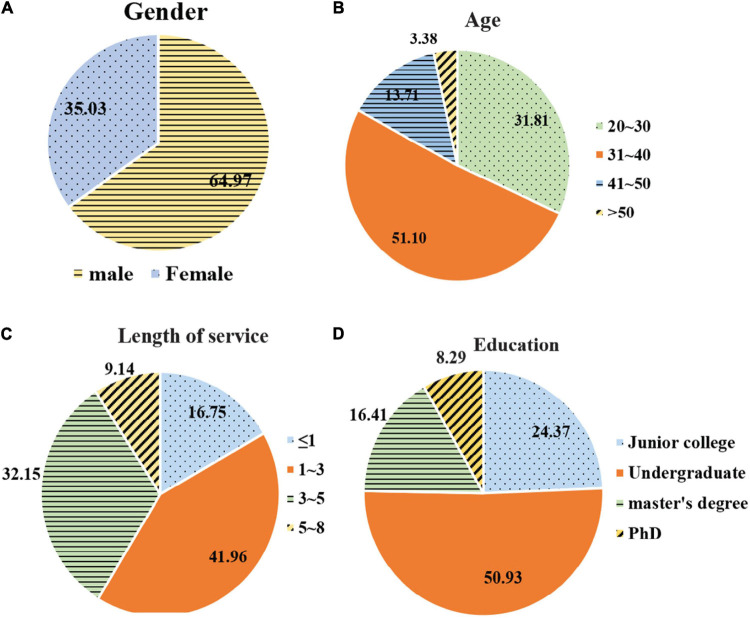
Basic characteristics of individuals. **(A)** Sex ratio; **(B)** Age distribution; **(C)** Seniority distribution; **(D)** Educational background.

There are 591 valid samples in this study. Of them, 384 are males (64.97%) and 207 are females (35.03%). The proportion of males is significantly higher than that of females (Figure 2A). There are 302 between 31 and 40 years old (51.10%), and 20 over 50 years old (3.38%) (Figure 2B). The number of people with working experience of 1–3 years is 248 (41.96%), and the number of people with working experience of 5–8 years is 54 (9.14%) (Figure 2C). Of 591 surveyed subjects, the number of people with a bachelor’s degree is 301 (50.93%), followed by a college degree 144 (24.37%), and a master’s degree 97 (16.41%), and there are only 49 (8.29%) with a doctoral degree (Figure 2D).

### Basic Characteristics of the Enterprise

**FIGURE 3:**
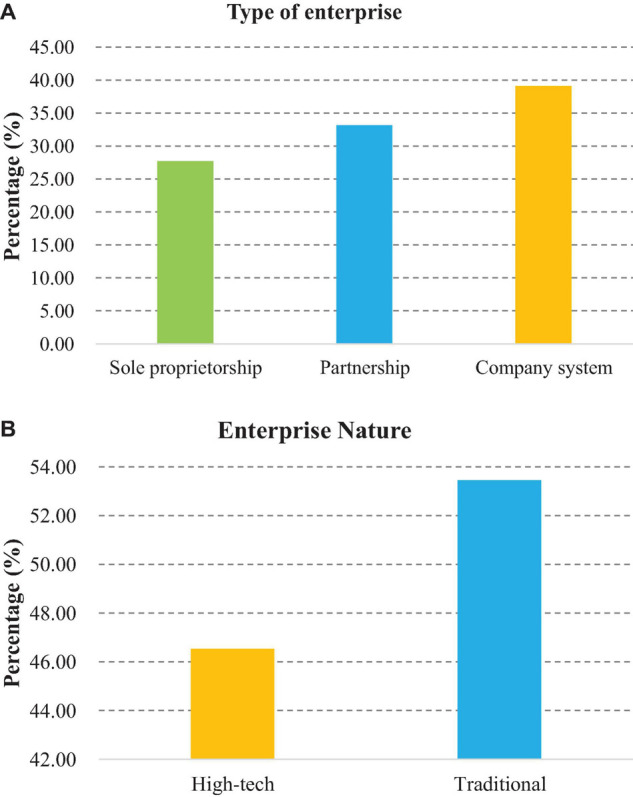
Type and nature of enterprises. **(A)** Type of enterprise; **(B)** Nature of enterprise.

As shown in Figure 3A, there are 56 sole proprietorship enterprises, accounting for 27.72%; there are 67 partnership enterprises, accounting for 33.17%; and there are 79 corporate enterprises, accounting for 39.11%. There are 108 traditional enterprises, accounting for 53.47%; and there are 94 high-tech enterprises, accounting for 46.53% (Figure 3B).

**FIGURE 4:**
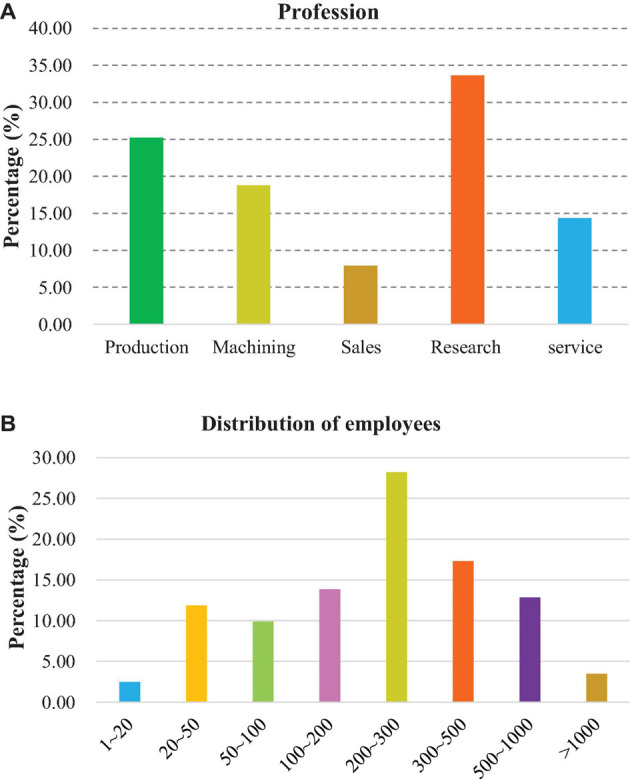
The belonging industry and number of employees. **(A)** The belonging industry; **(B)** Employee distribution.

According to the business statistics of enterprises, there are 68 R&D enterprises, accounting for 33.66%, followed by production enterprises, accounting for 25.25%. There are 38 processing enterprises, accounting for 18.81%. The number of the service and sales enterprises is 29 and 16, respectively, accounting for 14.36 and 7.92% (Figure 4A). As for the number of employees (Figure 4B), there are 35 companies with a number of employees between 200 and 300, accounting for the highest 10.33%; there are 28 companies with between 100 and 200 employees, accounting for 13.89%; there are seven companies with more than 1,000 employees, accounting for 3.47%, and there are five companies with less than 20 employees, accounting for 2.48%.

### Validity Analysis

Table 4 shows the factor loading values between work environment, innovation behavior, and corporate performance. The factor loading values of the three indicators are all higher than 0.7 (>0.4), and the average variance extracted (AVE) of all factors is greater than 0.5, indicating that the scale has good convergence validity. The significance probability of Bartlett’s sphericity test of each variable is 0.000, indicating that each variable is suitable for factor analysis.

**TABLE 4:** KMO and sphericity test.

	**KMO metrics**	**Bartlett’s sphericity test**		
		**Approximate Chi-Square distribution**	**Degree of freedom**	**Significance probability**
Teamwork	0.753	247.145	6	0.000
Organizational incentives	0.766	311.032	10	0.000
Superior support	0.812	626.126	12	0.000
Adequate resources	0.844	685.547	20	0.000
Work stress	0.769	241.194	7	0.000
Innovation of employees	0.922	1361.551	57	0.000
Innovation performance	0.817	672.958	30	0.000

### Factor Analysis of Work Environment Scale

**FIGURE 5:**
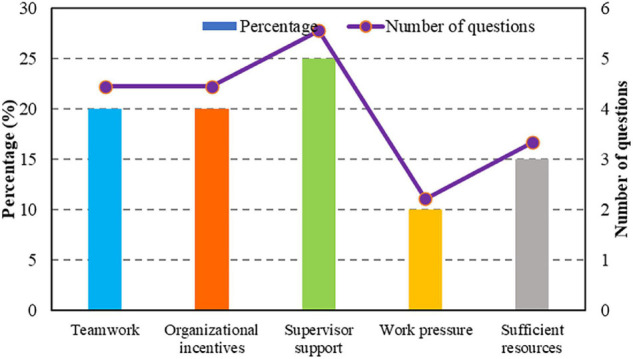
Factor analysis of enterprise work environment scale.

In this study, there are 18 questions in the enterprise work scale, including four questions in teamwork, innovative behavior, and organizational motivation, and the explicable variable ratio is 22.2%; 5 questions in leadership support, and the ratio of explanatory variables is 27.8%; two questions in work stress and three questions in sufficient resources, with explanatory variable ratios of 11.1 and 16.7% (Figure 5).

### Correlation Analysis

Table 5 shows the correlation between the work environment and the innovation behavior of employees. Teamwork, superior support, and work stress are all related to the innovation behavior of employees (*P* < 0.05), while organizational incentives and adequate resources are not related to innovation behavior (*P* > 0.05).

**TABLE 5:** Correlation analysis of enterprise work environment and employee innovation behavior.

**Factor**	**Correlation coefficient**	***P*-value**
Teamwork	0.29	0.003
Organizational incentives	0.07	0.242
Superior support	0.24	0.004
Adequate resources	0.15	0.636
Work stress	–0.16	0.003

Further, the hierarchical regression analysis is performed to verify the correlation between the factors in this study. The regression results of each factor (Table 6) reveal that there is no individual correlation coefficient (0.003–0.826) greater than its own reliability (0.684–0.966), indicating that the variables have a high degree of discrimination. The data meets the normal distribution, and the hypotheses can be tested.

**TABLE 6:** Statistical description and correlation coefficients between research variables.

	**Mean**	**Standard deviation**	**1**	**2**	**3**	**4**	**5**	**6**	**7**	**8**	**9**	**10**
1 Teamwork	5.221	1.022	0.740									
2 Superior support	5.125	1.125	0.582	0.921								
3 Work stress	5.641	1.043	–0.469	–0.405	–0.755							
4 Innovation behavior	5.194	0.912	0.551*	0.552*	0.463*	0.748						
5 Corporate performance	5.457	1.074	0.458*	0.194*	0.198*	0.211Δ	0.806					
6 Type of enterprise	5.229	1.328	0.328	–0.377	–0.276	–0.432	–0.094	–0.922				
7 Seniority	4.693	1.029	0.292	0.128	0.328	0.457	0.098	0.618	0.826			
8 Education background	3.625	1.662	0.446	–0.023	–0.079	–0.049	–0.144	–0.009	0.119*	1		
9 Age	3.351	4.292	0.005	0.035	0.022	0.053	0.029	0.089	0.237	0.228	1	
10 Sex	3.441	0.996	0.025	0.058	0.092	0.128	0.084	0.196	0.154	0.056	0.328	1

*Means significant at 95% confidence level, *P* < 0.05.

Δ*Means significant at 99% confidence level, *P* < 0.01.*

### Multiple Hierarchical Regression Model

The hierarchical regression analysis includes four models. Model 1 represents the regression between the control variables (teamwork, leadership support, and work stress) on the dependent variable (business performance); Model 2 represents the regression of independent variable (Employee innovation behavior) on dependent variable (corporate performance) on the basis of Model 1; Model 3 represents the regression of the moderating variable (work environment) on the employee innovation behavior on the basis of Model 2; Model 4 represents the regression of adjustment variable (redundant resources and innovation culture) on employee innovation behavior and service innovation performance (Table 7).

**TABLE 7:** Hierarchical regression analysis.

**Variables**	**Model 1**	**Model 2**	**Model 3**	**Model 4**
Gender	–0.125	–0.029	–0.497	–0.044
Age	–0.442	–0.045	–0.613	–0.068
Education background	–0.038	–0.072	–0.711	–0.071
Seniority	–0.049	–0.063	–0.084	–0.057
Type of enterprise	–0.218	–0.032	–0.056	–0.036
Innovation behavior		0.419Δ	0.124*	0.136*
Teamwork	0.219Δ	0.194Δ	0.228Δ	0.214Δ
Superior support	0.226Δ	0.225*	0.145Δ	0.135Δ
Work stress	0.192Δ	0.135Δ	0.122*	0.193*
Work environment × innovation behavior				0.032Δ

*Means significant at 95% confidence level, *P* < 0.05.

Δ*Means significant at 99% confidence level, *P* < 0.01.*

### Hypothesis Test Result

As for model 1, its *R* value is 0.628, *R*^2^ value is 0.392, and ΔR^2^ value is 0.217, indicating that the model fits well. It shows that superior support, teamwork, and work stress in the work environment are all correlated with employee innovation behavior (*P* ≤ 0.01), and the corresponding hypothesis is established. In Model 2, the *R* value is 0.568, the *R*^2^ value is 0.334, and the ΔR^2^ value is 0.283, indicating that employee innovation behavior is significantly positively correlated with corporate performance (β = 0.375, *P* ≤ 0.01), so the hypothesis is valid. In Model 3, its *R* value is 0.511, *R*^2^ value is 0.196, and ΔR^2^ value is 0.172, which indicates that work environment and employee innovation behavior promote enterprise performance (β = 0.433, *P* ≤ 0.01), so the hypothesis is valid. In Model 4, the *R* value is 0.652, the *R*^2^ value is 0.416, and the ΔR^2^ value is 0.337, indicating that the work environment promotes the relationship between employee innovation behavior and corporate performance (β = 0.399, *P* ≤ 0.05), so the hypothesis is valid. The specific results are shown in Table 8.

**TABLE 8:** Hierarchical regression analysis results.

**Hypothesis**	**Result**
Organizational encouragement in the work environment is significantly related to employee innovation behavior	Invalid
Superior support in the work environment is significantly correlated with employee innovation behavior	Valid
Teamwork has a significant correlation with employee innovation behavior	Valid
Adequate resources in the work environment have a significant correlation with employee innovation behavior	Invalid
Work stress is significantly correlated with employee innovation behavior	Valid
Employee innovation behavior positively affects corporate performance	Valid
The work environment has a significant correlation with corporate performance	Invalid
Work environment and employee innovation behavior promote corporate performance	Valid
The work environment promotes the relationship between employee innovation behavior and corporate performance	Valid

## Discussion

The results of this study show that leadership support, teamwork, and work stress in the work environment are significantly correlated with employee innovation behavior. Among them, leadership support and teamwork in the work environment are significantly positively related to employee innovation behavior, and work stress is significantly negatively correlated with employee innovation behavior. It suggests that these three factors will have a greater impact on employee innovation behavior. This is similar to the results of the study (Eder et al., 2020; Anastas and Zimmerman, 2021). Further analysis of the correlation between work environment and corporate performance shows that there is no significant correlation between the two, but employee innovation behavior can promote corporate performance, so work environment can promote the relationship between employee innovation behavior and corporate performance to a certain extent. Hence, to promote the innovation behavior of employees, it first needs to build a good work environment. A good work environment is conducive to the creativity of members (Cva et al., 2020). That enterprises encourage employees and take risks that may arise from innovation strengthens employees’ safe psychological awareness toward problem solving (Wu W. et al., 2020). Teamwork encourages employees to communicate, and depends on the accessibility of information flow (Gong et al., 2020), and teamwork is more conducive to the completion of creative tasks. Superior support gives employees a space for autonomy and creativity (Shen et al., 2019; Gucyeter and Erdogan, 2021). A relaxed environment is more conducive to their active participation in the work decision-making process.

In the study, it is found that the regression coefficient of employee innovation behavior to corporate performance is 0.375, and there is a significant correlation between the two (*P* ≤ 0.01). It suggests that employee innovation behavior is significantly positively correlated with corporate performance. Researchers have investigated the relationship between employee innovation behavior and performance from the perspectives of role theory and social learning theory, and the results show that employee innovation behavior can significantly affect employee performance. Wei et al. (2020) conducted a survey on private enterprises in Turkey and found that employees’ participation in innovation can improve the efficiency of work roles and departments, and the improvement of performance can increase the competitiveness and success rate of employees. Hoque et al. (2017) proposed that expected performance is significantly correlated with innovation behavior. Some research results indicated that employee innovation behavior has a significant impact on task performance (Li C. et al., 2020). It is believed that innovation behavior obviously promotes corporate performance. Li L. et al. (2020) pointed out that employee innovation behavior has a significant impact on task performance. Chang (2020) perform found that employee innovation behavior is significantly positively correlated with the work efficiency and performance of the department. Studies have pointed out that employee innovation behavior can improve work efficiency and corporate performance, while increasing employee competitiveness (Ibidunni et al., 2020). In this study, new ventures are used as the research subjects, and the correlation between employee innovation behavior and corporate performance is analyzed. In practice, in order to improve corporate performance and market share, the new venture needs to provide a good work environment, and actively encourage employees to carry out innovative activities and stimulate their innovation behavior. Employee innovation behavior positively affects corporate performance, and the implementation of employee innovation activities is inseparable from the support of organizations and groups. Therefore, the new venture should learn from major companies and actively encourage employees to carry out innovative activities. A good innovation environment and positive incentive policies should be provided for employees as much as possible to inspire their innovation behavior, and ultimately promote the business performance.

## Conclusion

In this study, the impact of work environment and employee innovation behavior on the performance of a new venture is analyzed based on personality psychology. It is found that the work environment of new venture has a significant impact on employee innovation behavior, that employee innovation behavior positively affects corporate performance, and that the work environment promotes the relationship between employee innovation behavior and corporate performance. However, this research still has some shortcomings. This research does not further analyze the correlation between corporate innovation culture and redundant resources with employee innovation behavior, which should be discussed in the follow-up to strengthen the findings of the study. In conclusion, this research provides useful guidance and reference for the development of new ventures in China.

## Data Availability Statement

The raw data supporting the conclusions of this article will be made available by the authors, without undue reservation.

## Ethics Statement

The studies involving human participants were reviewed and approved by the Changshu Institute of Technology Ethics Committee. The patients/participants provided their written informed consent to participate in this study. Written informed consent was obtained from the individual(s) for the publication of any potentially identifiable images or data included in this article.

## Author Contributions

All authors listed have made a substantial, direct and intellectual contribution to the work, and approved it for publication.

## Conflict of Interest

The authors declare that the research was conducted in the absence of any commercial or financial relationships that could be construed as a potential conflict of interest.

## Publisher’s Note

All claims expressed in this article are solely those of the authors and do not necessarily represent those of their affiliated organizations, or those of the publisher, the editors and the reviewers. Any product that may be evaluated in this article, or claim that may be made by its manufacturer, is not guaranteed or endorsed by the publisher.

## Abbreviations

KMO: Kaiser-Meyer-Olkin
SPSS: statistical product and service solutions.
